# Extensively Drug-Resistant (XDR) Typhoid: Evolution, Prevention, and Its Management

**DOI:** 10.1155/2020/6432580

**Published:** 2020-05-02

**Authors:** Javed Akram, Arsalan Shamim Khan, Hassan Ahmed Khan, Syed Amir Gilani, Shehla Javed Akram, Fridoon Jawad Ahmad, Riffat Mehboob

**Affiliations:** ^1^Department of Physiology, University of Health Sciences, Lahore, Pakistan; ^2^National University of Sciences & Technology (NUST), Islamabad, Pakistan; ^3^Abbottabad University of Science and Technology, Abbottabad, Pakistan; ^4^Faculty of Allied Health Sciences, The University of Lahore, Lahore, Pakistan; ^5^Akram Medical Complex, Lahore, Pakistan

## Abstract

Typhoid fever is the result of a human host-restricted *Salmonella enteric* serotype typhi infection that causes enteric fever. Around 21 million people contract typhoid annually, with Pakistan's inhabitants at most risk amongst Asian countries where typhoid remains prevalent. Decades of indiscriminate antibiotic usage has driven the evolution of multidrug-resistant strains and more recently, extensively drug-resistant (XDR) strains of *Salmonella enteric* serotype typhi. Current reports of extensively drug-resistant typhoid fever outbreak in Pakistan are not only a major concern for Pakistan but also for health authorities worldwide: intercontinental transmission, spread, and replacement of native strains in neighboring countries and a major impediment to Pakistani health care management. The WHO records that there are 5274 cases of extensively drug-resistant (XDR) typhoid fever out of a total of 8188 total cases of typhoid fever reported in Pakistan. The last remaining feasible oral antibiotic that XDR typhoid remains susceptible to is azithromycin; this is a cause of major concern. Additionally, several cases of XDR typhoid fever have also been reported in patients travelling from Pakistan to the USA, UK, and Canada. This review article attempts to raise the issue of XDR typhoid with respect to its epidemiology, prevention, management, and future outlook and stresses a better understanding of antimicrobial stewardship and general surveillance of the disease. Although progress is being made to combat XDR typhoid locally, efficient, unified efforts on a national and international scale are required to contain the XDR outbreak before it is no longer manageable and leads us back to the preantibiotic era.

## 1. Introduction

*Salmonella enterica* serotype typhi (*Salmonella typhi*) exists as a gram-negative, rod-shaped, flagellated bacterium. It features a polysaccharide capsule which confers virulence to the bacterium by providing protection against phagocytosis [1]. Serological testing identifies positively for antigens O9 and O12 in the polysaccharide component of its surface lipopolysaccharide (LPS), as well as for virulence (Vi) capsular polysaccharide [2]. The only host for *Salmonella typhi* are humans in whom it causes typhoid fever, characterized by a progressively rising, life-threatening fever, in conjunction with various clinical symptoms.

Although years of research have led to a better general understanding of the disease, much remains unknown. The monophyletic structure of *Salmonella* typhi suggests that it is a relatively new disease [3] and is thought to have evolved 50,000 years ago [4]. With the first descriptions of typhoid fever dating back to the early 1800s, the discovery of the causative pathogen, *Salmonella typhi*, was made in 1880 by Karl Eberth [5].

Despite major advances in health care and medicine, millions across the globe are at risk of contracting typhoid and paratyphoid fever as a consequence of exposure to the causative organism, leading to disabilities and even death [6]. As opposed to the risk of fatality between 10 and 30% in the preantimicrobial era, cases presenting typhoid fever today have a risk of fatality of less than 1%. At present, a major threat to effective management of typhoid fever is the emergence of *Salmonella typhi* strains that are resistant to antimicrobial agents [7]. The term MDR described resistance to ampicillin, trimethoprim-sulfamethoxazole, and chloramphenicol while XDR exhibit resistance to chloramphenicol, ampicillin, co-trimoxazole, and fluoroquinolones, as well as third-generation cephalosporins.

Several indicators including the incidence, morbidity, mortality, and financial costs involved with typhoid fever need to be evaluated in paving the way for the successful management and/or eradication of enteric fever. These data, in conjunction with local economy and resources, are essential to take appropriate public health measures. Additionally, understanding the trends associated with the disease are also important in providing informed guidance to travelers. [8]

## 2. Global Epidemiology of Typhoid Fever

Global control over typhoid presents a serious threat to public health. Typhoid fever is the result of enteric infection caused by *Salmonella typhi*. Concurrently, *Salmonella enterica* serovar *paratyphi* A, B, and C (*S. paratyphi*) are responsible for causing paratyphoid fever. In 2015, 17 million global cases of typhoid and paratyphoid fever were reported collectively. Of these, the greatest bioburden and incidence had been in South Asia, among Southeast Asia, and sub-Saharan Africa. Untreated cases of typhoid and paratyphoid fever have been reported to have led to 178,000 deaths globally in 2015 [9].

More recent reports suggest that around 21 million contract typhoid annually, leading to 161,000 cases ending in fatality. Inhabitants of the Pakistani provinces of Punjab and Sindh have been declared to be most at risk of developing typhoid, out of all of the 16 Asian countries where typhoid is prevalent [10].

## 3. Antimicrobial Usage and XDR Typhoid

The discovery of novel antibiotics against typhoid fever in the past has saved millions of lives across the planet. Unfortunately, decades of antibiotic usage have driven the evolution of multidrug-resistant and extensively drug-resistant strains of typhoid fever.

### 3.1. Evolution of Antimicrobial Resistance

Prior to the advent of antibiotics, typhoid fever was a real challenge for health care practitioners owing to its chances of relapse, carriage, and complications. In 1948, chloramphenicol emerged as an antimicrobial remedy to combat enteric fever caused by typhoidal salmonella. Two years later, however, clinical reports indicating the organism's resistance against chloramphenicol began. As the 1970s approached, evidence of horizontal transfer of resistance genes in *Salmonella typhi* was brought to the attention of researchers. As a consequence, a now chloramphenicol-resistant variant of *Salmonella typhi* had taken over the globe. During the 1970s, around 10,000 cases of MDR typhoid strains were reported in Mexico during an epidemic. Eventually, the epidemic was contained and although sporadic events of MDR cases were reported in other countries, these never became dominant.

For the next decade, ampicillin and trimethoprim–sulfamethoxazole, in spite of their possibly inferior efficacy as opposed to earlier therapeutic agents (also known as co-trimoxazole), became the sought-after antimicrobial drugs for treating typhoid. Around the early 1990s, multidrug-resistant typhoid strains exhibiting resistance to all three antibiotics began to plague the world in conjunction with a rise in fatality rates. For the next 2 decades, fluoroquinolones, especially ciprofloxacin, were employed across the globe as the preferred line of treatment for typhoid.

**Figure 1:**
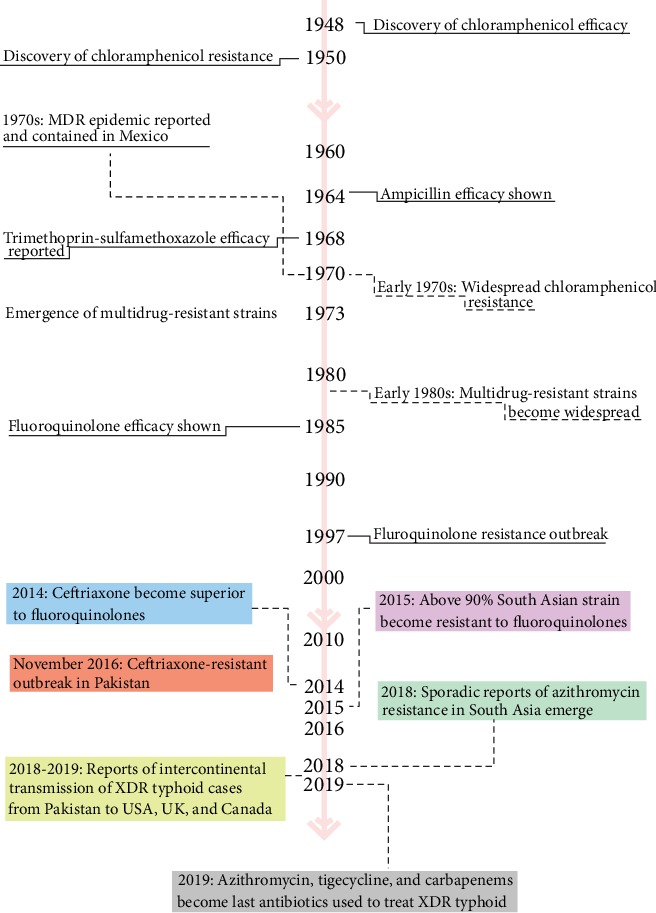
History of antibiotic efficacy studies and the emergence of antimicrobial resistance in *Salmonella typhi*.

Recent years, however, have seen the rise of high-level fluoroquinolone-resistant infections, which form the majority of typhoid cases in South Asia and threatens to spread globally. As a result, clinicians have turned towards the use of azithromycin and cephalosporins as the last lines of treatment for which there exists supporting clinical trial evidence. Today, in addition to the bioburden of enteric fever already present in South Asia, there is evidence of an emergence of a cephalosporin-resistant strain of *Salmonella typhi*. Although the first outbreak has been reported in Sindh, it is not just a concern for Pakistan, as cephalosporin-resistant strains of *Salmonella typhi* have also been recorded in India, Bangladesh, Philippines, Iraq, and Guatemala [11–13].  Figure 1describes a brief recollection of major events pertaining to antimicrobial resistance in *Salmonella typhi*.

### 3.2. Evolution from Multidrug-Resistant (MDR) to Extensively Drug-Resistant (XDR) Typhoid

Recent years have seen the global spread of a frequently multidrug-resistant (MDR) haplotype of *Salmonella typhi*, called H58, which is common in Asia and some parts of Africa. MDR typhoid traditionally describes resistance to all the first line of antibiotics suggested by WHO: ampicillin, trimethoprim-sulfamethoxazole, and chloramphenicol. In Pakistan, MDR and quinolone-resistant strains of *Salmonella typhi* pose great health risks. Although MDR *S. typhi* is declining, quinolone-resistant strains continue to be ubiquitous. A study at Aga Khan University, Pakistan, from 2001 to 2006 suggests that the multidrug resistance rate for *Salmonella typhi* strains has gone up from 34.2% to 48.5%, while resistance against quinolone has shot up from 1.6% to 64.1% through these years [14, 15].

Following the emergence of a fluoroquinolone-resistant *Salmonella typhi* strain, the choice of treatment has been third-generation cephalosporins, such as ceftriaxone. Following November 2016, Sindh has seen the rise of ceftriaxone-resistant cases of typhoid fever. It is suggested that *Salmonella typhi* possesses the ability to transform from MDR to XDR by simply acquiring a plasmid, conferring its resistance against all recommended lines of treatment. Thus, it is suggested that this particular H58 clade is responsible for the outbreak, with its XDR variant harboring an IncY plasmid, providing resistance against fluoroquinolone, as well as the CTX-M-15 gene *bla*, and protecting the organism against ceftriaxone [16]. It remains to be seen which other resistance the organism might acquire as a result of antibiotic stewardship. Current treatment options for Pakistani MDR and XDR strains of *Salmonella typhi* are provided in Table 1.

### 3.3. Distribution of Extensively Drug-Resistant (XDR) Typhoid in Pakistan

In November 2016, the WHO records suggest that Pakistani health authorities began reporting cases of extensively drug-resistant (XDR) typhoid fever, originating in Hyderabad, Sindh. These reports, from November 2016 up to December 2018, have recorded a total of 5274 cases of XDR typhoid fever, out of a total of 8188 cases of typhoid fever.

**Figure 2:**
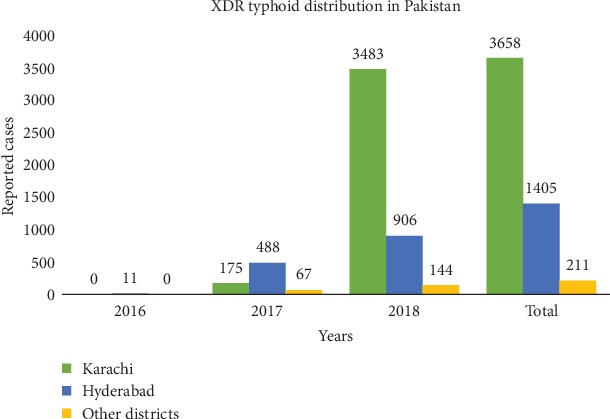
Extensively drug-resistant typhoid distribution in Pakistan. This is a graphical representation of data reproduced from WHO disease outbreak news [17].

The capital city of Sindh, Karachi, accounted for 69% of all cases of XDR. Hyderabad district recorded 27% of these cases while the remaining 4% were distributed amongst other provincial districts of Sindh. The distribution of these cases from 1 November, 2016, to 09 December, 2018, is shown in Figure 2 [17].

### 3.4. Intercontinental Transmission of Extensively Drug-Resistant (XDR) Strains of *Salmonella typhi* from Pakistan

Reports from January to October, 2018, also indicate the international transmission of XDR strains of *Salmonella typhi* associated with travelers to Pakistan. Of the total six such cases, one was reported in the United Kingdom of Great Britain and Northern Ireland, while the remaining five were reported in the United States of America. Reports suggest that four of the six cases reported here had either travelled to or were residents of Karachi (Sindh), Lahore (Punjab), or Islamabad.

Further breakdown suggests that the cities of Karachi, Lahore, and Islamabad were the common destinations for two of these cases. For the other two travel-associated cases, one is a resident of Lahore while the other has travel history to Karachi. Evidence suggests that all travel-associated cases of XDR typhoid strain were successfully treated. However, information regarding exposure details and time on disease onset is unknown [17]. Recently, a case of XDR typhoid fever has been successfully diagnosed and treated in Canada. The child in question had travelled to Sindh, returned to Canada where the diagnosis was made, and was treated with intravenous meropenem [18].

The CDC notes that everyone travelling to Pakistan is at risk of contracting XDR typhoid strain, with those planning family visits being at more risk than those travelling to the country for business and tourism. The CDC also declares a level 2 alert with respect to the outbreak whereby enhanced precautions need to be practiced [19].

## 4. Prevention

*Salmonella typhi* infections are restricted only to human hosts and are passed on through contaminated water supplies and bad hygiene practices, such as through fecal contamination [20]. The pathogen's acquisition of a host and consequent infection may be averted if certain practical prevention measures may be considered.

### 4.1. Immunization

Since the processes of rapid urbanization in developing countries present great barriers to the complete eradication of contamination from water supplies, immunization against typhoid provides the best route of protection from typhoid. As of 2018, the WHO has not only prequalified a tetanus-toxoid conjugated Vi polysaccharide typhoid vaccine developed in India (Typbar TVC), but it has also suggested the prioritized usage of such a vaccine in counties where typhoid is endemic and exhibits antimicrobial resistance [21]. Gavi, the vaccine alliance, has committed $85 million towards the production of conjugate vaccines in poverty-stricken countries [11]. An estimated 250,000 children in Hyderabad have received the WHO prequalified Vi-polysaccharide typhoid vaccine since 2017 [10].

### 4.2. Hygiene and Water Supply

As had been the case with North America and Europe before the 20^th^ century, typhoid remained an epidemic concern until measures ensuring adequate sanitation and the distribution of treated water were employed. The relevant governmental authorities in developing countries should thus ensure the education of the general public about good hygiene practices, as well as the infrastructural operations required to bring about the eradication of human waste from water supplies. [22]

### 4.3. Food Safety

Food that is cooked thoroughly may generally be regarded as safe because of the high temperatures lowering the survivability of microbes. It should be warned, however, that if the same food is allowed to sit for extended periods of time at room temperature, then, it may run the risk of becoming contaminated again. Dry food, packaged in factories with good hygiene practices, may also be regarded as safe for consumption, as any microorganisms present would need moisture for their survival. Conversely, raw foods, including meats, fruits, and vegetables, as well as street food should be avoided as the risk of contamination is high. Similarly, tap water, fresh juices, fountain drinks, and ice from unknown sources in developing countries are best avoided as well, as these may have come to be products of a contaminated water supply. [23]

## 5. Professional Health Care Management

**Figure 3:**
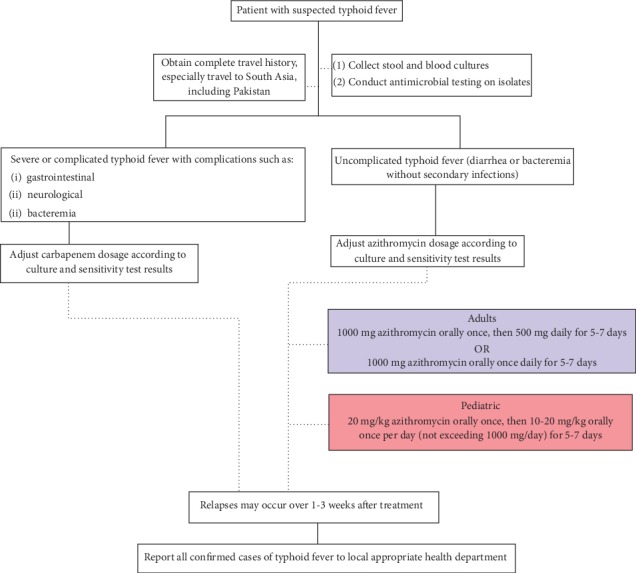
Treatment plan against XDR typhoid fever for health care professionals as suggested by CDC [19].

During the course of treatment of typhoid cases from Pakistan, two things should be noted. First, it should be understood that the Pakistani strain of XDR typhoid exhibits susceptibility to azithromycin and carbapenems. Secondly, about 90% of *Salmonella typhi* isolates from Pakistan have low susceptibility to or exhibit resistance against fluroroquinolones, including ciprofloxacin. Therefore, ciprofloxacin may not be effective for typhoid sufferers who have acquired the illness from Pakistan. The flowchart in Figure 3 attempts to aid health care providers in caring for typhoid fever cases.

With ever-decreasing treatment options for typhoid fever, XDR typhoid presents itself as a grave concern that commands serious attention before it becomes a global health concern. Reports indicate decreased susceptibility to azithromycin [24], which clinicians are using as a last resort for treatment for XDR typhoid fever, along with tigecycline and carbapenems [12].

Carbapenems are expensive, and their mainstream use is unfavorable given the resource-stricken environments where typhoid is most prevalent. Moreover, both tigecylcine and carbapenems are administered parenterally, and the concurrently rising resistance of typhoid against azithromycin highlights an additional need to develop orally administered antibiotics. Since 90% of typhoid patients are treated as outpatients, a limited number of oral antibiotic options would necessitate treating such cases as inpatients, thereby improving the chances of over burdening hospitals where nosocomial infections associated with drug-resistant pathogens are common.

Additionally, carbapenems resistance has already been witnessed in nontyphoidal *salmonella* serovars and thus, as evolution predicts, *Salmonella* typhi may not be too slow to acquire such resistance. Since the global-scale dissemination of such a resistant microorganism would be swift, the WHO identifies Salmonellae as a target for the development of novel antibiotics [11].

## 6. Future Outlook

Health authorities across the globe should be warned that the XDR H58 haplotype of *Salmonella typhi* originating in Sindh, a densely populated area in Asia, has the capacity to invade and spread to regions beyond Pakistan, with the potential to replace native strains [25]. The 6 reports of travel-associated international transmission of XDR typhoid to the United States and the United Kingdom, as well as the first Canadian pediatric case of XDR typhoid, well demonstrate its ability to spread intercontinentally.

Local health care providers are cautioned by the fact that the prospective acquisition of resistance to azithromycin by XDR typhoid presents a single step that leaves them with a pathogen which is practically untreatable in a developing country such as Pakistan. This predicts a scenario where approximately 15% of typhoid cases in developing countries end in fatality [26].

Fortunately, the only long-term reservoir for *Salmonella typhi* is humans; no zoonotic reservoirs exist and the bacterium only persists in the environment for as long as the carrier sheds it into the surroundings by fecal material which may last from months to years. Environmental adaptations, such as spore-formation, are absent in the bacteria, and so, it possesses only a limited capacity to survive long-term [12].

Informal settlement areas lacking proper sanitation and adequate water infrastructure facilitate the transmission and international migration of XDR typhoid strain from Pakistan across the rest of the globe. Thus, practical recommendations to contain the local outbreak continues to be the taking up of adequate sanitation measures by local government and wide spread immunization by the typbar TVC vaccine. For the former of these recommendations, according to news reports, the provincial government has set aside 399 million Pakistani rupees for the restoration of water distribution networks and 414 million Pakistani rupees for the revival of the sewage system in Hyderabad [10].

A national-scale XDR typhoid task force, approved by the WHO, was formed in July 2018. Educational campaigns raising awareness about XDR typhoid and the importance of good hygiene habits are a need of the hour. Campaigns that target the locals' concerns regarding foreign vaccines and their consequent refusal of immunization also need to be addressed, as vaccinators face refusal regularly. The WHO advises that microbiological tests, including antimicrobial susceptibility tests, be conducted on suspected typhoid fever patients to confirm the presence of *Salmonella typhi*, as well as to determine its resistance. It also suggests that any emerging resistance be closely monitored by increased surveillance.

There remains a direct need for global efforts to come together to produce novel antibiotics against *Salmonella* infections which continue to plague developing countries. Although antibiotics save countless lives annually, their efficacy is challenged by the ease and swiftness with which pathogens such as *Salmonella typhi* can acquire resistance. The current state of antimicrobial surveillance in Pakistan is poor and needs urgent attention to both understand and predict future outcomes in antimicrobial resistance, and thus to allow for appropriate antibiotic stewardship if we are to prevent ourselves from going back to the preantibiotic era.

## Figures and Tables

**Table 1:** Treatment options for MDR and XDR typhoid in Pakistan.

Antibiotic	Route of administration	Typhoid susceptibility	
		MDR	XDR
Chloramphenicol	Oral, intravenous	No	No
Co-trimoxazole	Oral, intravenous	No	No
Ampicillin	Oral, intramuscular, intravenous	No	No
Ciprofloxacin	Oral, intravenous	Yes	No
Ceftriaxone	Intramuscular, intravenous	Yes	No
Azithromycin	Oral	Yes	Yes
Meropenem	Intravenous	Yes	Yes
Tigecycline	Intravenous	Yes	Yes

MDR: multidrug resistance; XDR: extensively drug-resistant.
